# Proteomic Analysis Reveals CACN-1 Is a Component of the Spliceosome in *Caenorhabditis elegans*

**DOI:** 10.1534/g3.114.012013

**Published:** 2014-06-19

**Authors:** Michael F. Doherty, Guillaume Adelmant, Alyssa D. Cecchetelli, Jarrod A. Marto, Erin J. Cram

**Affiliations:** * Biology Department, Northeastern University, Boston, Massachusetts 02115; † Department of Cancer Biology and Blais Proteomics Center, Dana-Farber Cancer Institute, Boston, Massachusetts 02215; ‡ Department of Biological Chemistry and Molecular Pharmacology, Harvard Medical School, Boston, Massachusetts 02115

**Keywords:** proteomics, cell migration, *C. elegans*, spliceosome

## Abstract

Cell migration is essential for embryonic development and tissue formation in all animals. *cacn-1* is a conserved gene of unknown molecular function identified in a genome-wide screen for genes that regulate distal tip cell migration in the nematode worm *Caenorhabditis elegans*. In this study we take a proteomics approach to understand CACN-1 function. To isolate CACN-1−interacting proteins, we used an *in vivo* tandem-affinity purification strategy. Tandem-affinity purification−tagged CACN-1 complexes were isolated from *C. elegans* lysate, analyzed by mass spectrometry, and characterized bioinformatically. Results suggest significant interaction of CACN-1 with the *C. elegans* spliceosome. All of the identified interactors were screened for distal tip cell migration phenotypes using RNAi. Depletion of many of these factors led to distal tip cell migration defects, particularly a failure to stop migrating, a phenotype commonly seen in *cacn-1* deficient animals. The results of this screen identify eight novel regulators of cell migration and suggest CACN-1 may participate in a protein network dedicated to high-fidelity gonad development. The composition of proteins comprising the CACN-1 network suggests that this critical developmental module may exert its influence through alternative splicing or other post-transcriptional gene regulation.

Cell migration is critical for development of the embryo, gastrulation, and formation of the nervous system (Lauffenburger and Horwitz 1996). Deregulated cell migration contributes to autoimmune disease and metastatic cancer (Ridley *et al.* 2003). To study cell migration *in vivo*, we are using gonad morphogenesis in *Caenorhabditis elegans* as a model system. The *C. elegans* gonad tissue develops from a group of four cells known individually as Z1, Z2, Z3, and Z4 (Hubbard and Greenstein 2000). The distal cells of the gonad primordium, Z1 and Z4, give rise to the somatic components of the gonad tissue. The central cells, Z2 and Z3, give rise to the germ line (Kimble and Hirsh 1979). The development of the gonad is controlled by two specialized leader cells, the distal tip cells (DTCs), which are derived from Z1 and Z4 during the L1 stage (Kimble and White 1981; Nishiwaki 1999; Lehmann 2001). At the onset of the second larval stage (L2), the DTCs begin to migrate in opposite directions, and in response to attractive and repulsive cues execute turns to form a U-shaped, symmetrical tube (Wong and Schwarzbauer 2012). DTC migration occurs in three distinct phases. The DTCs migrate along the ventral basement membrane (commencement), take a 90° turn toward the dorsal side of the animal and then make another 90° turn back toward the mid-body (turning), and then migrate along the dorsal basement membrane until they have reached the mid-body point opposite the vulva, where they stop (cessation) (Wong and Schwarzbauer 2012).

Migrating DTCs respond to long- or short-range matrix and diffusible cues. These cues activate conserved cell signaling pathways and ultimately impinge upon the cytoskeleton. For example, proper DTC migration relies on proteolytic-mediated remodeling of the extracellular matrix (Kawano *et al.* 2009; Ismat *et al.* 2013), signaling through the small GTPases (Lundquist *et al.* 2001), Netrin chemical gradients (Ziel and Sherwood 2010), and integrin signaling (Baum and Garriga 1997; Nikolopoulos and Giancotti 2005; Meighan and Schwarzbauer 2007). Because the DTCs are the leader cells responsible for the correct formation of the gonad, failure of the DTC to migrate correctly results in the formation of a misshaped organ. Thus, gonad formation provides a facile *in vivo* model to examine the genetic controls required to coordinate proper cell path finding and orientation during migration.

In a previous genome-wide RNA interference (RNAi) screen for animals with DTC migration defects (Cram *et al.* 2006) we identified CACN-1 as a well-conserved and novel regulator of cell migration. This screen was designed to identify genes with a strong and highly penetrant role in DTC migration. CACN-1 is expressed in the DTC throughout development and is required within the DTC for normal turning and cessation of migration (Tannoury *et al.* 2010). CACN-1 has three isoforms, and genetic analysis suggests that the primary isoform, CACN-1A, is responsible for regulating DTC migration (Tannoury *et al.* 2010). CACN-1A contains a nuclear localization signal at its N-terminus, two coiled-coil domains, and a well-conserved C-terminal Cactus domain (Schultz *et al.* 2000; Kosugi *et al.* 2009).

CACN-1/Cactin was first identified in Drosophila as a cactus binding protein with roles in dorsal-ventral polarization during fly embryogenesis (Lin *et al.* 2000). In humans, Cactin (C19ORF29) was originally described as a renal carcinoma antigen (Scanlan *et al.* 1999) and more recently as a negative regulator of Toll-like receptor and innate immune signaling (Atzei *et al.* 2010). In the parasite, *Toxoplasma gondii*, Cactin acts as a multimer to moderate gene expression by regulating AP2 transcription factors (Szatanek *et al.* 2012). Evidence is accumulating that Cactin may play a role in splicing or post-transcriptional regulation of gene expression. For example, in *Arabidopsis thaliana*, *CACTIN* is a nuclear protein required for embryogenesis and colocalizes with spliceosomal components (Baldwin *et al.* 2013), and human Cactin has been isolated with the C complex of spliceosome (Jurica *et al.* 2002; Bessonov *et al.* 2008, 2010; Ilagan *et al.* 2009; Ashton-Beaucage *et al.* 2014).

The spliceosome, the machinery that is responsible for the removal of introns and the ligation of exons, is composed of >100 proteins (Zahler 2012). Pre-mRNA splicing increases the variety of gene transcripts and provides a mechanism to regulate gene expression post transcriptionally (Wollerton *et al.* 2001). The spliceosome is a dynamic complex composed of the pre-mRNA, four small ribonucleoprotein particles (snRNPs), and many other associated factors (Chen and Cheng 2012). The snRNPs themselves are multicomponent complexes, composed of protein and snRNA, responsible for catalysis in the mature C complex of the spliceosome (Wahl *et al.* 2009). Many accessory proteins are also required for spliceosomal activity, including ATP-dependent DexD/H-box RNA helicases (Riddle and Blumental 1997; Rocak and Linder 2004), SR proteins, and other nucleic acid binding proteins (Blencowe 2000). In *C. elegans*, many of these splicing-associated factors remain uncharacterized.

To elucidate the mechanism by which CACN-1 mediates cell migration in *C. elegans*, we used tandem affinity purification and mass spectrometry to identify CACN-1−interacting molecules. We found that CACN-1 copurified with multiple subunits of the *C. elegans* spliceosome. Using an RNAi-based functional assay, we found that many proteins associated with CACN-1 are required for proper DTC migration.

## Materials and Methods

### Generation of tandem affinity purification (TAP) vector (hsp16.2 > flag::ha::cacn-1a)

The *cacn-1a* ORF was amplified using reverse-transcription polymerase chain reaction from *C. elegans* mRNA using primers with *Pst*I 5′ extensions and ligated into the pUC19 MCS at *Pst*I. The *hsp16.2* promoter was amplified from pPD49_83 (Andrew Fire, Stanford University) using primers with *Kpn*I 5′ extensions and ligated into the pUC19 (Invitrogen, Carlsbad, CA) at *Kpn*I (5′ to *cacn-1* cDNA::*cacn-1* 3′UTR). The *flag*::*ha* cassette was amplified from pOZ-N (a gift from Y. Nakatani, Dana-Farber Cancer Institute) and was recombined 5′ of *cacn-1* cDNA::*cacn-1* 3′UTR using InFusion HD (Clontech, Mountain View, CA) at *Kpn*I. Primer and plasmid details are available upon request.

### Nematode strains and generation of transgenic animals

Nematodes were cultivated on nematode growth media (NGM) agar plates with *Escherichia coli* OP50 bacteria following standard techniques (Brenner 1974). Nematode culture and observations were performed at 23°, unless otherwise indicated. Young adult N2 hermaphrodites were injected using a microINJECTOR apparatus (Tritech Research, Los Angeles, CA). The injection solution contained 20 ng/µL transgene, 20 ng/µL *sur-5*::*gfp*, and 60 ng/µL N2 genomic DNA digested with *Dpn*I (New England Biolabs, Ipswich, MA). The green fluorescent protein marker allowed tracking and maintenance of the transgenic line, UN1305 *xbEx1305[hsp16.2p*::*flag*::*ha*::*cacn-1*, *sur-5*::*GFP]*. Because N2 is used as a negative control in the TAP-MS experiments, nontransgenic siblings of *xbEx1305*-expressing animals are not expected to increase background.

### Growth of nematodes and induction of recombinant protein expression

Starved plates were chunked to 10-cm NGM agar plates seeded with *E. coli* strain OP50. Animals were propagated as an asynchronous population at 23° for approximately 5 d or until the animals had consumed the majority of their bacterial food source. To induce the expression of *hsp16.2p*::*flag*::*ha*::*cacn-1*, plates were transferred from 23° to 33° for 4 hr. Animals were removed from the incubator and placed at room temperature for 2 hr to recover from the heat shock.

### Nematode lysis

Postheat shock, animals were collected with M9-Tween (22 mM KH_2_PO_4_, 42 mM NaHPO_4_, 86 mM NaCl, 1 mM MgSO_4_, 0.1% Tween-20), and allowed to settle and consume remaining bacteria for approximately 30 min. Animals were then collected by centrifugation and washed twice with M9-Tween to remove remaining bacteria, and resuspended in lysis buffer (50 mM Tris pH 7.5, 150 mM NaCl, 1 mM ethylenediaminetetraacetic acid, 0.5% v/v NP-40, 10% v/v glycerol, 1X HALT Protease Inhibitor; Thermo). Animals were vortexed briefly and submerged in liquid nitrogen, thawed quickly at 42°, and refrozen in liquid nitrogen. Lysate was produced by grinding the frozen slurry with a pestle in a 35-mm diameter mortar suspended in liquid nitrogen until a fine powder was obtained. The mortar was then removed from the liquid nitrogen, and allowed to thaw on ice. Thawed material was collected and stored on ice. The mortar was then washed twice with lysis buffer to capture remaining lysate. Lysate was clarified by centrifugation and stored immediately at −80°.

### Tandem FLAG-HA immunoprecipitation

The tandem affinity purification strategy is adapted from Nakatani and Ogryzko (2003). Protein loading was normalized to total protein between the two conditions. Lysate was batch-bound to anti-FLAG agarose using lysis buffer (50 mM Tris pH 7.5, 150 mM NaCl, 1 mM ethylenediaminetetraacetic acid, 0.5% v/v NP-40, 10% v/v glycerol (Merck KGaA), 1X HALT Protease Inhibitor.) Bound proteins were washed with lysis buffer and competitively eluted using lysis buffer supplemented with 250 µg/mL FLAG peptide. FLAG eluates were batch-bound to anti-HA agarose. Bound proteins were washed with lysis buffer and competitively eluted using lysis buffer supplemented with 200 µg/mL HA peptide.

### Sodium dodecyl sulfate-polyacrylamide gel electrophoresis (SDS-PAGE) and Western analysis

SDS-PAGE was performed by the method of Laemmli (1970) using Mini-PROTEANTGX 10% acrylamide gels (Bio-Rad, Hercules, CA) and stained using a silver-stain protocol (Switzer *et al.* 1979) to enhance the visibility of low abundance proteins. Gels were transferred to 0.2-µm Whatman nitrocellulose membranes (GE Healthcare, Buckinghamshire, UK) in a Transblot Cell (Bio-Rad) (Towbin *et al.* 1979). We used a mouse-anti-FLAG primary antibody (Rockland Immunochemicals, Gilbertville, PA) diluted 1:1,000 to detect FLAG::HA::CACN-1. Bands were visualized using a donkey-anti-mouse secondary antibody (1:10,000) coupled to horseradish peroxidase and subsequent ECL treatment.

### Protein digestion

Proteins from the TAP samples were directly processed in solution. Cysteine residues were reduced with 10 mM DTT for 30 min at 56° and alkylated with 22.5 mM iodoacetamide for 20 min at room temperature in the dark. Proteins were digested overnight at 37° using 5 μg of trypsin after adjusting the pH to 8.0 with 1 M Tris. The resulting tryptic peptide solutions were acidified by adding trifluoroacetic acid to a final concentration of 1% and desalted by C18 solid phase extraction followed by strong cation exchange, both performed in batch-mode format. Eluted peptides were concentrated in a vacuum concentrator, and reconstituted with 20 µL of 0.1% trifluoroacetic acid.

### Liquid chromatography–mass spectrometry/mass spectrometry analysis

Purified tryptic peptides were analyzed by liquid chromatography–mass spectrometry/mass spectrometry (Ficarro *et al.* 2009) on an LTQ-Orbitrap-XL mass spectrometer (Thermo, Waltham, MA) equipped with a Digital PicoView electrospray source platform (New Objective, Woburn, MA). The spectrometer was operated in data-dependent mode where the top nine most abundant ions in each MS scan were subjected to CAD (35% normalized collision energy, isolation width = 2.8 Da, threshold = 20,000). Dynamic exclusion was enabled with a repeat count of 1 and exclusion duration of 30 sec. ESI voltage was set to 2.2 kV.

MS spectra were recalibrated using the background ion (Si(CH3)2O)6 at m/z 445.12 +/− 0.03 and converted into a Mascot generic file format (.mgf) using multiplierz scripts (Askenazi *et al.* 2009; Parikh *et al.* 2009). Spectra were searched using Mascot (version 2.3) against three appended databases consisting of: i) *C. elegans* protein sequences (WS220); ii) common lab contaminants; and iii) a decoy database generated by reversing the sequences from these two databases. For Mascot searches, precursor tolerance was set to 1 Da and product ion tolerance to 0.6 Da. Search parameters included trypsin specificity, up to 2 missed cleavages, fixed carbamidomethylation (C, +57 Da) and variable oxidation (M, +16 Da). Precursors with a recalibrated mass error greater than 10 ppm were discarded. Spectra matching to peptides from the reverse database were used to calculate a global false discovery rate, and were discarded. Data were further processed to remove peptide spectral matches to the forward database with an FDR greater than 1.0%.

### Protein network analysis

Protein−protein interactions were analyzed and visualized using Pathway Palette (Askenazi *et al.* 2010). Interaction networks were abstracted as graphs, where genes are represented as nodes and their interactions as edges. The 20 genes identified as CACN-1 interactors were uploaded to the Pathway Palette website (Askenazi *et al.* 2010). Known interactions between these genes were retrieved from a database of known and predicted interactions assembled by Raymond Lee, Sternberg Lab, Caltech (personal communication). To further explore connectivity between proteins within the CACN-1 network, interactions from the aforementioned database were retrieved to construct a network extended to first-order neighbors connecting two or more CACN-1 interactors. To assess the physical cohesiveness of the resulting network, we implemented physical interaction enrichment (PIE) analysis (Sama and Huynen 2010) using a custom Python script and a reference network consisting of the 20,465 protein-coding genes with 28,386 known or predicted interactions between them, as represented in Pathway Palette. In total, 10,000 selected gene sets, having the same size and node degree distribution as the extended CACN-1 graph (containing 27 genes), were generated to obtain a frequency distribution of number of edges observed in random networks. This distribution was used to calculate the physical cohesiveness (PIE score) of the CACN-1 network and its significance.

### RNAi

Starved nematodes were allowed to recover on fresh OP50 seeded NGM plates for 2 d. This procedure produces gravid young adult hermaphrodites for egg collection. Eggs were released using alkaline hypochlorite solution (Hope 1999). Following two washes in M9 buffer (22 mM KH_2_PO_4_, 42 mM NaHPO_4_, 86 mM NaCl, 1 mM MgSO_4_), eggs were transferred to plates seeded with RNAi HT115(DE3) bacteria expressing dsRNA. For all RNAi experiments, the wild-type nematode strain N2 was used. RNAi was performed by feeding the animals *E. coli* strain HT115(DE3) expressing gene-specific dsRNA (Timmons *et al.* 2001). The RNAi feeding protocol was essentially as described by Timmons and Fire (1998). Bacteria transformed with the dsRNA construct of interest were cultured overnight in LB supplemented with 40 µg/mL ampicillin. The following day, 150 μL of culture was seeded onto NGM agar supplemented with carbenicillin (25 µg/mL) and 1 mM isopropyl β-D-1-thiogalactopyranoside. Double-stranded RNA expression was induced overnight at room temperature on the isopropyl β-D-1-thiogalactopyranoside plates. Embryos generated by alkaline lysis were then transferred onto the plates and the RNAi phenotypes were monitored at the times indicated.

### Analysis of RNAi phenotypes

Adult nematodes were harvested at 65 hr postalkaline lysis preparation to analyze DTC migration defects. Animals were mounted in a drop of M9 containing 0.08 M sodium azide on a slide coated with 2% agarose in water and imaged by differential interference contrast microscopy using a 60X oil-immersion objective with a Nikon Eclipse 80i epifluorescence microscope equipped with a SPOT RT3 CCD camera (Diagnostic Instruments; Sterling Heights, MI). DTC migration defects were inferred from the resulting shape of the gonad arms. Defects such as insufficient distance migrated along the ventral surface, inappropriate or extra turns, and failure to cease migrating at the vulva were counted as DTC migration defects. Images were captured using SPOT Advanced version 4.6.4.6 software (Diagnostic Instruments or Axiovision software, Release 4.5). DTC migration defects between treatments were statistically compared using the Fisher’s exact test (two-dimensional χ^2^ analysis) using GraphPad Prism statistical software.

## Results

### Tandem affinity purification of CACN-1 complexes

**Figure 1:**
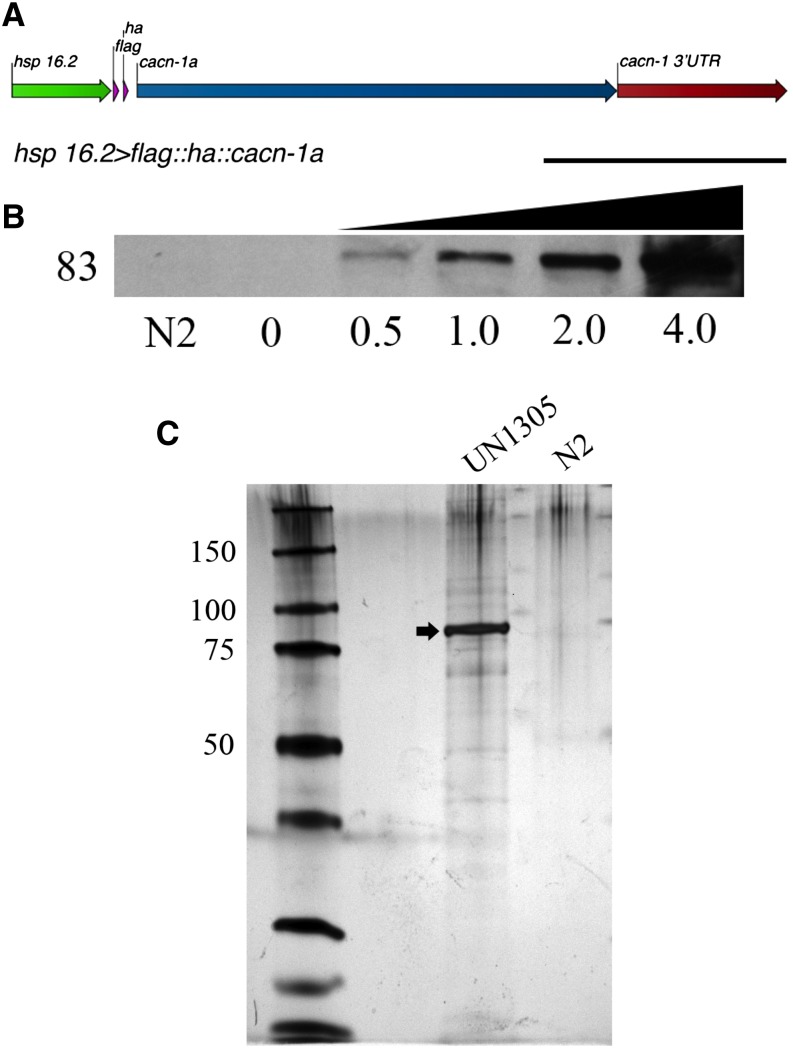
Expression of FLAG::HA::CACN-1 is induced by heat shock and multiple proteins co-purify with FLAG::HA::CACN-1. (A) The genetic construct used to create the transgenic nematode line UN1305 is composed of a 400-bp heatshock promoter (*hsp 16.2)*, the FLAG::HA tandem affinity tag, a short undecapeptide linker, the *cacn-1a* open reading frame, and the *cacn-1* 3′ untranslated region (UTR). Scale bar = 1 kb. (B) Western blot analysis of protein lysates prepared from UN1305 after a time course of heat shock at 33° indicates significantly increased expression after the given number of hours of heat shock treatment. Induction of FLAG::HA::CACN-1 was detected with an anti-FLAG antibody at the expected molecular weight of 83 kDa. (C) Silver-stained sodium dodecyl sulfate polyacrylamide gel electrophoresis analysis of coimmunoprecipitates with FLAG::HA::CACN-1 following TAP. As a negative control, wild-type (N2) lysate was processed through TAP to establish a background of non-specific interactions. The arrow indicates FLAG::HA::CACN-1.

To identify CACN-1−interacting proteins, we designed and expressed a tagged version of CACN-1 optimized for TAP. A short linker peptide (GGLEGTRGSSS) separated the N-terminal bipartite FLAG::HA tag from full-length CACN-1A (Figure 1A). The FLAG::HA dual affinity tag is commonly used in TAP experiments and offers high specificity and good yields (Nakatani and Ogryzko 2003; Adelmant *et al.* 2012; Rozenblatt-Rosen *et al.* 2012; Andrawes *et al.* 2013; Kim *et al.* 2014; Watanabe *et al.* 2014). The *hsp16.2* promoter drives expression of the transgene in most tissues of the worm with strongest expression in the intestine and pharynx (Link *et al.* 1999). *hsp16.2*::FLAG::HA::CACN-1A is strongly expressed in animals subjected to heat shock (Figure 1B).

For TAP, mixed-stage *C. elegans* expressing *hsp16.2*::FLAG::HA::CACN-1A were incubated at 33° for 4 hr to induce strong expression of CACN-1 and subsequently harvested. Nontransgenic (N2) animals served as a negative control. Lysates prepared from both genotypes were subjected to sequential FLAG and HA affinity purifications (Adelmant *et al.* 2012). An aliquot of the final eluates was analyzed using SDS-PAGE silver stain to visualize proteins that specifically coprecipitated with CACN-1 (Figure 1C). The remaining eluates from two independent biological replicates were analyzed by mass spectrometry (*i.e.*, tandem affinity purification mass spectrometry), generating a list of putative CACN-1−interacting proteins (Supporting Information, Table S1).

### CACN-1 is part of an interconnected protein network

**Figure 2:**
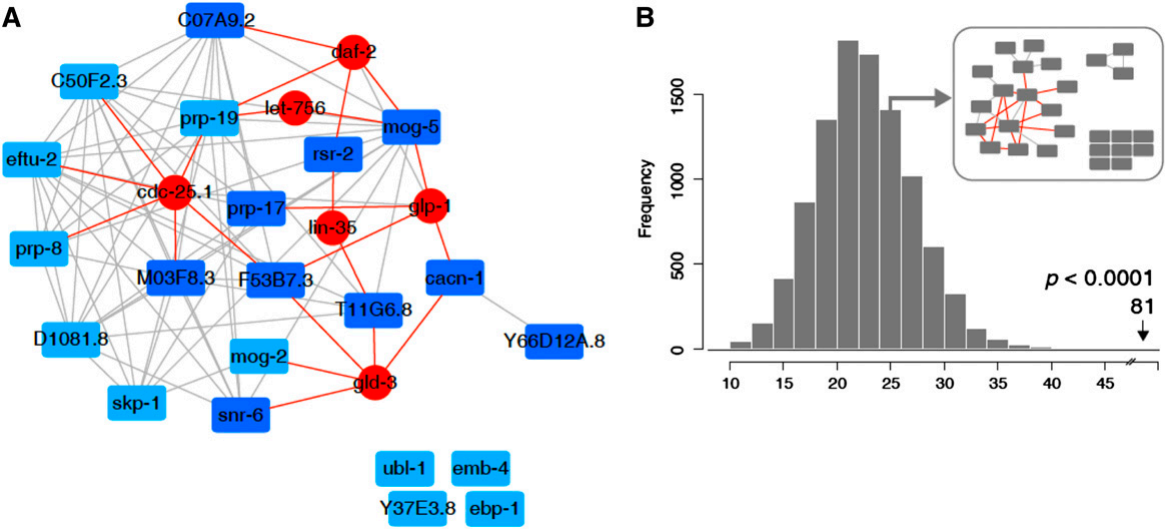
Analysis of the CACN-1 protein interaction network. Known and predicted interactions among proteins associated with CACN-1 were analyzed and visualized by Pathway Palette. The resulting network displays significant cohesiveness. (A) The 20 proteins identified as CACN-1 interactors (blue nodes) were used as input to retrieve known and predicted (gray edges) interactions. Known, first degree interactors of these genes, were retrieved from the WS234 database and added to the network. Only those connected to two or more CACN-1 interactors were kept in the final graph (red edges and nodes). CACN-1 interactors that displayed significant DTC migration defects when depleted with RNAi are colored dark blue. (B) Edge frequency distribution was computed by generating 10,000 random graphs selected to exhibit the same number of nodes and degree distribution. The position indicating the number of edges (81) observed in the CACN-1 network is indicated by the arrow, along with the p-value. An example random graph is shown in the inset. Additional information about each node can be found in Table 2.

The list of candidate CACN-1 interactors was filtered to remove proteins identified in only one of the two independent TAP experiments, found in the negative control N2 animals, heat shock proteins, or proteins found in organelles such as the mitochondria. After filtering, 20 proteins remained. We next used Pathway Palette (Askenazi *et al.* 2010) to query Wormbase and retrieve experimentally based genetic, physical and regulatory interactions among the 20 proteins identified in our TAP-MS analysis. We also interrogated a database of predicted interactions based on a study from Zhong and Sternberg (2006). A majority of these proteins, with the exception of CACN-1, formed a highly connected component based on predicted interactions (Figure 2A). To connect CACN-1 to this central component, we expanded our network by querying Wormbase to retrieve additional proteins known to interact with members of the core network. To enforce network parsimony we retained only those proteins that exhibited two or more interactions with its members. This analysis added six protein nodes and twenty edges (Figure 2A and Table 1). Two of these additional proteins, GLP-1 and GLD-3, have known interactions with CACN-1 and with some of its interacting partners thereby bridging CACN-1 and the rest of the network. To evaluate the overall connectivity of this network, we used the recently developed PIE analysis whereby 10,000 random networks were generated using nodes with the same degree distribution as observed in the CACN-1 network (Sama and Huynen 2010; Meixner *et al.* 2011). This analysis yielded a PIE score of 2.16, indicating that the CACN-1 network is indeed significantly more connected than a network of similar size derived from genes selected at random (*P* < 0.0001, Figure 2B).

**Table 1:** Proteins predicted to interact with two or more binding partners of CACN-1

*C. elegans* Sequence ID	Locus	*H. sapiens* Gene	BLAST	Similarity (Identity)*^a^*	Functional Class	Description
C05D11.4	*let-756*	FGF20	6.9E-24	63% (42%)	Signaling	FGF-like ligand
C32F10.2	*lin-35*	RBL2	2.4E-45	42% (25%)	Signaling	Retinoblastoma protein homolog
F02A9.6	*glp-1*	NOTCH1	1.1E-88	39% (26%)	Signaling	Notch receptor
K06A5.7	*cdc-25.1*	CDC25.A	2.0E-24	57% (36%)	Cell cycle	CDC25 phosphatase homolog
T07F8.3	*gld-3*	BICC1	9.0E-06	44% (20%)	Nucleic Acid binding	KH domain RNA-binding protein
Y55D5A.5	*daf-2*	INSR	2.7E-143	54% (37%)	Signaling	Insulin/IGF receptor homolog

Data derived from Wormbase WS241. FGF, fibroblast growth factor; IGF, insulin-like growth factor.

a Within homologous region.

### CACN-1 associates with spliceosomal proteins

**Figure 3:**
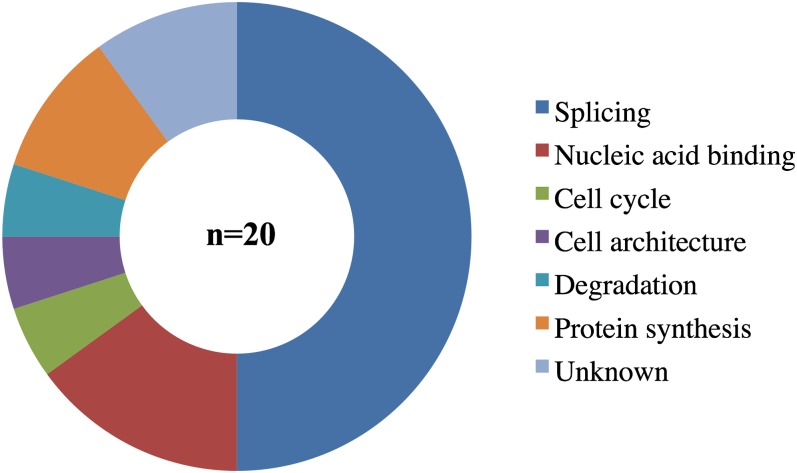
The majority of CACN-1−interacting proteins are splicing factors. Previously assigned functional classes (Kamath *et al.* 2003) were used to categorize each CACN-1−interacting protein. The functional class “Nucleic acid binding” includes the categories: RNA binding, DNA binding, NA binding, and chromatin.

There is growing evidence that biomolecules within physical, genetic, or metabolic networks often share common biological function (Balázsi *et al.* 2011; Vidal *et al.* 2011). Consistent with this idea, we found that a majority (11/20) of the proteins in copurified with CACN-1 have been characterized in *C. elegans* as splicing components (Figure 3 and Table 2). Others are involved with cellular processes such as the cell cycle, degradation, protein synthesis, and cell architecture (Figure 3 and Table 2). Some of these proteins have homology to nucleic acid binding proteins and could be involved with pre-mRNA splicing; others are uncharacterized. Human homologs were retrieved by querying Wormbase WS238. All of the CACN-1 interactors show significant similarity to human proteins (Table 2), suggesting that the CACN-1 complex is evolutionarily well conserved. To determine whether the homologs are associated with the human spliceosome, we identified the closest human homologs of the identified CACN-1 interacting proteins (Table 2) and queried the Spliceosome Database (Spliceosome Database) database, a compendium of results derived from analysis of the human spliceosome. Most of these orthologs have been identified in mass spectrometric analyses of native human spliceosomes (Table 3), and generally associate with the late catalytic conformation of the spliceosome rather than with the early spliceosome. All of the proteins are associated with the catalytically active C complex.

**Table 2:** CACN-1−interacting proteins

*C. elegans* Sequence ID	Locus	Unique Peptides Generated	*H. sapiens* Gene	BLAST	Similarity (Identity)*^a^*	Functional Cass	Description
C07A9.2		3	BUD31	5.9E-60	86% (69%)	Nucleic acid binding	Unknown
C50C3.6	*prp-8*	28	PRPF8	0	91% (84%)	Splicing	U5 snRNP
C50F2.3		12	XAB2	3.8E-223	71% (51%)	Splicing	Splicing factor
D1081.8		9	LY6D	1.3E-06	63% (48%)	Cell cycle	Cell division cycle 5-like protein
EEED8.5	*mog-5*	6	DHX8	0	77% (65%)	Nucleic acid binding	DEAH helicase
F49D11.1	*prp-17*	8	CDC40	8.2E-166	62% (37%)	Splicing	Splicing factor
F53B7.3		3	ISY1	1.0E-62	56% (41%)	Splicing	Splicing factor
H06104.4	*ubl-1*	4	RPS27A	4.2 e-43	76% (50%)	Degradation	Ubiquitin-like protein
H20J04.8	*mog-2*	5	SNRPA1	2.4E-56	72% (55%)	Splicing	U2 snRNP
M03F8.3		17	CRNKL1	4.3E-242	80% (61%)	Splicing	Crooked neck-like protein
T10F2.4	*prp-19*	9	PRPF19	1.4E-136	67% (50%)	Splicing	Splicing factor
T11G6.8		3	RBM22	2.0E-116	76% (60%)	Splicing	Pre-mRNA splicing factor
T27F2.1	*skp-1*	11	SNW1	2.0E-153	68% (53%)	Nucleic acid binding	SKI-binding protein
Y37E3.8		3	RPL27A	3.9 e-56	70% (58%)	Protein synthesis	60S ribosomal protein
Y49E10.15	*snr-6*	4	SNRPE	3.9E-56	86% (66%)	Splicing	snRNP E
Y57A10A.19	*rsr-2*	12	SRRM2	3.4E-45	65% (43%)	Unknown	SR protein
Y59A8B.7	*ebp-1*	2	MAPREI	1.3 e-50	58% (42%)	Cell architecture	Microtubule (+) binding protein
Y66D12A.2		8	CXorf56	2.6E-34	51% (35%)	Unknown	Unknown
Y80D3A.2	*emb-4*	18	AQR	0	68% (53%)	Splicing	Intron-binding splicesomal protein ’Aquarius’
ZK328.2	*eftu-2*	12	EFTUD2	0	80% (67%)	Protein synthesis	Elongation factor

Data derived from Wormbase WS241. snRNP, snRNPs.

a Within homologous region.

**Table 3:** Homologs of CACN-1−interacting proteins associate with the late spliceosome

*C. elegans*		*H. sapiens*		
Sequence name	Locus	Gene Name	Class/Family	Splicesomal Complex Association(s)
W03H9.4	*cacn-1*	CACTIN	Recruited at C complex	C complex, P complex
C07A9.2		BUD31	PRP19 related	B complex, B^act^ complex, C complex, P complex
C50C3.6	*prp-8*	PRPF8	U5 snRNP	B complex, B^act^ complex, C complex, U5 snRNP, tri-snRNP, P complex
C50F2.3		XAB2	PRP19 related	B complex, B^act^ complex, C complex, P complex
D1081.8		LY6D		
EEED8.5	*mog-5*	DHX8	Second step factors	C complex, P complex
F49D11.1	*prp-17*	CDC40	Second step factors	B^act^ complex, C complex, P complex
F53B7.3		ISY1	PRP19 related	B complex, B^act^ complex, C complex, P complex
H06104.4	*ubl-1*	RPS27A		
H20J04.8	*mog-2*	SNRPA1	17S U2 snRNP	17S U2 snRNP, A complex, B complex, B^act^ complex, C complex, P complex
M03F8.3		CRNKL1	PRP19 related	B complex, B^act^ complex, C complex, P complex
T10F2.4	*prp-19*	PRPF19	PRP19 complex	B complex, B^act^ complex, C complex, P complex
T11G6.8		RBM22	PRP19 related	B complex, B^act^ complex, C complex, P complex
T27F2.1	*skp-1*	SNW1	PRP19 related	B complex, B^act^ complex, C complex, P complex
Y37E3.8		RPL27A		
Y49E10.15	*snr-6*	SNRPE	Sm complex subunit	17S U2 snRNP, A complex, B complex, B^act^ complex, C complex, U1 snRNP, U4/U6 snRNP, U5 snRNP, tri-snRNP, P complex
Y57A10A.19	*rsr-2*	SRRM2	SR related	C complex, P complex
Y59A8B.7	*ebp-1*	MAPREI		
Y66D12A.8		CXorf56	Recruited at C complex	C complex, P complex
Y80D3A.2	*emb-4*	AQR	PRP19 related	B complex, B^act^ complex, C complex, P complex
ZK328.2	*eftu-2*	EFTUD2	U5 snRNP	B complex, B^act^ complex, C complex, U5 snRNP, tri-snRNP, P complex

Homology-based modeling suggests CACN-1 contains two coiled-coil domains. These domains often mediate interactions with other coiled-coil containing proteins (Burkhard *et al.* 2001). We identified coiled-coil domains present in the CACN-1−interacting proteins using SMART (Schultz *et al.* 2000). The CACN-1 interactors EBP-1, EMB-4, F53B7.3, M03F8.3, MOG-5, PRP-17, PRP-19, and SKP-1 each possess at least one coiled-coil domain, which may enable a direct interaction between CACN-1 and these proteins. In summary, these results are consistent with earlier studies in other species (Jurica *et al.* 2002; Baldwin *et al.* 2013), suggesting that CACN-1 associates with the spliceosome, perhaps through interactions mediated by the coiled-coil domains.

### CACN-1 interactors regulate DTC migration

**Figure 4:**
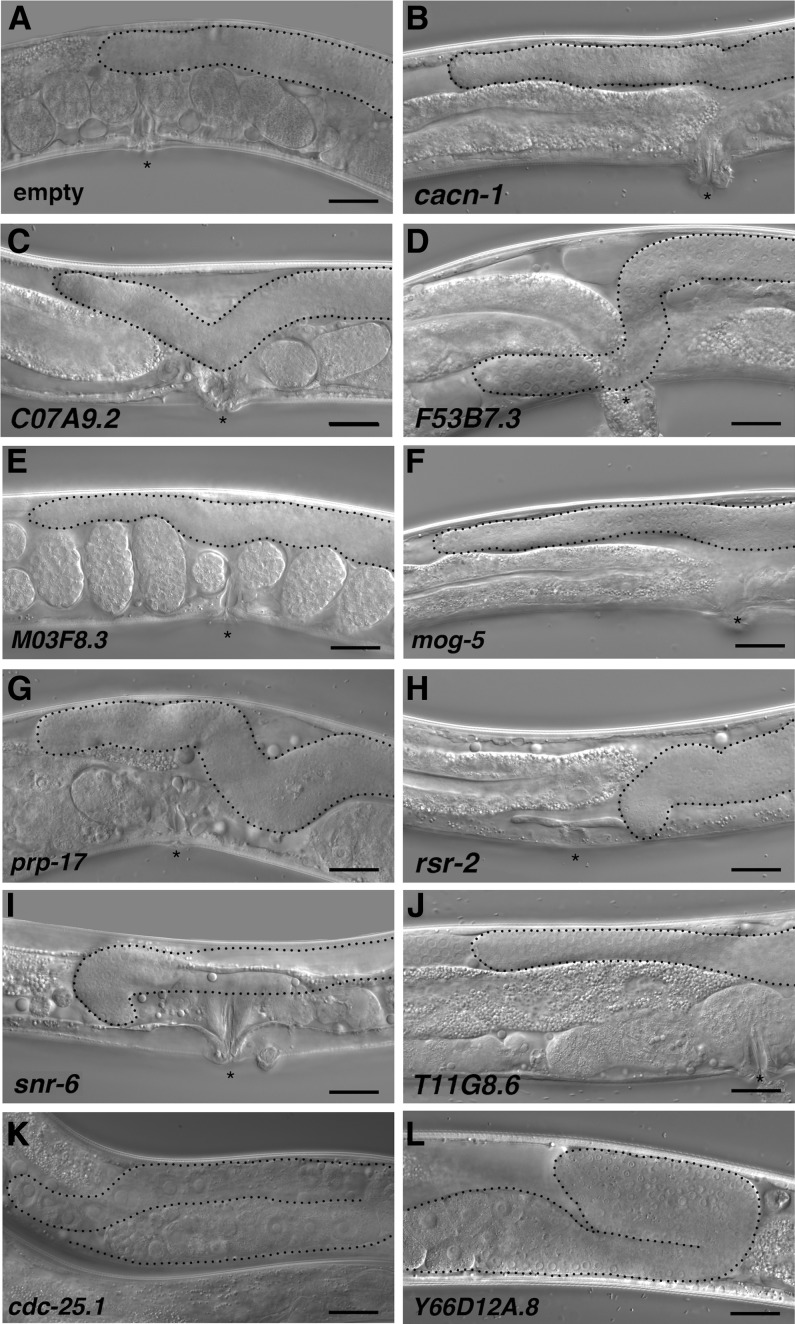
CACN-1−interacting proteins regulate distal tip cell migration *in vivo*. Wild-type (N2) animals were treated with each of the RNAi constructs described in (A−K). DTC migration and gonad arm morphology were assayed in young adults with the use of differential interference contrast microscopy. Representative DIC images are shown for (A) normal control animals and (B−J) nematodes treated with the indicated RNAi clones. These animals display the overshoot and “wandering” phenotypes. (K) Nematodes treated with Y66D12A.8 RNAi display a distal tip cell “early-stop” phenotype. Asterisks mark the vulva in adult animals (except for K and L, in which the vulva is just to the left of the image crop). Gonad arms are outlined in black. Scale bar = 25 microns.

**Figure 5:**
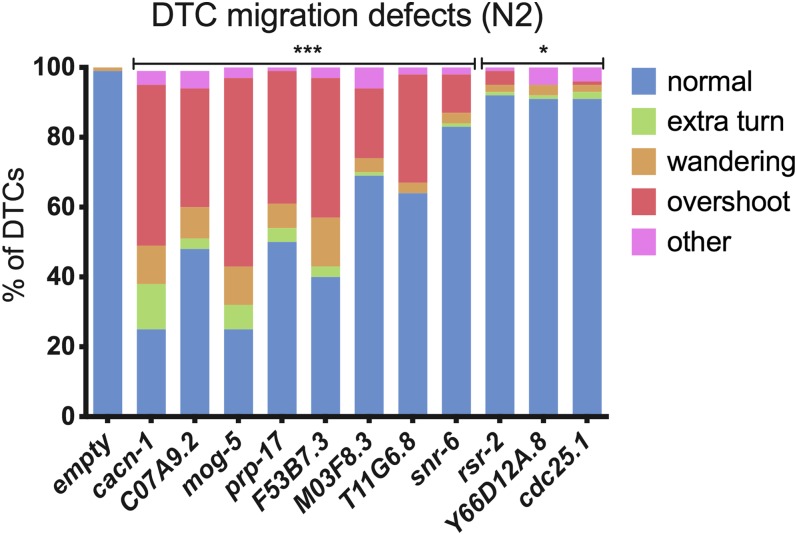
CACN-1−interacting proteins are required for distal tip cell migration. Distal tip cell migration defects such as incorrect navigational decisions (extra turns, wandering), failure to stop at the correct location (overshoot), and other defects, which include early failures in migration along the ventral surface were scored in young adult animals using DIC microscopy. Details are in Table 4. Fisher exact tests (two-dimensional χ^2^ analyses) were used to compare the % of normal gonad arms between empty RNAi treatment and each experimental condition. Statistical significance is denoted as: ***P < 0.0005; *P < 0.05.

We next used RNAi depletion to determine whether the proteins that co-purified with CACN-1 play a functional role in DTC migration. Normally, the two DTCs migrate in opposite directions along the ventral surface, turn toward the dorsal side, and then turn again to migrate back toward the midline, resulting in a mirror-image U-shaped gonad with each DTC at the midline (Figure 4A). The most prominent DTC migration phenotype in *cacn-1* RNAi animals is one in which the cells fail to stop migrating at the end of L4, bypassing the correct stopping point opposite of the vulva (Tannoury *et al.* 2010) (Figure 4B). If CACN-1−binding partners are required to orchestrate the transition of the DTC from a migratory to a stationary cell type at the end of larval morphogenesis, then depletion of these proteins should also result in the overshoot phenotype. To test this idea, animals were reared on bacterial lawns expressing dsRNA corresponding to each CACN-1 interactor. DTC migration defects were scored in adult animals using differential interference contrast microscopy. In control young adult animals, the DTC is correctly positioned at the midline (Figure 4A). We observed that genetic depletion of 9 of the 20 CACN-1 interactors displayed varying degree of DTC migration defects compared to nematodes treated with empty vector RNAi (Table 4 and Figure 4). Only one of these, *prp-17*, has been previously identified as a regulator of DTC migration (Cram *et al.* 2006). Similar to animals treated with *cacn-1* RNAi, the most common phenotype observed in each RNAi treatment was failure to stop migrating at the correct position (Figure 5). Several of the other CACN-1 interactors (*ubl-1*, *Y37E3.8*, *eftu-2*, *prp-8*, and *skp-1*) are required for general larval development, and nematodes treated with these RNAi constructs arrested as early-stage larvae, precluding scoring of the later phases of DTC migration. The 6 additional proteins identified as potential factors coupling the function of CACN-1 to cell signaling events were also scored for DTC migration defects. Only one of these factors, the protein phosphatase *cdc-25.1*, produced a statistically significant DTC migration defect, consisting mainly of mild navigational defects (Figure 4K and Figure 5). Depletion of proteins by feeding RNAi is not always sufficient to result in phenotypes (Simmer *et al.* 2002; Ahringer 2006); therefore, it is possible other CACN-1 interactors also play a role in DTC migration. The identification of eight novel regulators indicates that this network plays an important and previously unappreciated role in the regulation of DTC migration. In addition, it suggests that the proteins we identified are not spurious interactors, but work with CACN-1 *in vivo* to regulate cessation of DTC migration at the end of larval development.

**Table 4:** Quantification of dtc migration defects

Sequence name	Locus	normal)*^a^*	extra turn*^b^*	wandering*^c^*	overshoot*^d^*	other*^e^*	n
Control		99%	0%	1%	0%	0%	102
W03H9.4	*cacn-1*	25%	13%	11%	46%	4%	114
C07A9.2		48%	3%	9%	34%	5%	96
EEED8.5	*mog-5*	25%	7%	11%	54%	3%	100
F49D11.1	*prp-17*	50%	4%	7%	38%	1%	102
F53B7.3		40%	3%	14%	40%	3%	91
M03F8.3		69%	1%	4%	20%	6%	108
T11G6.8		64%	0%	3%	31%	2%	104
Y49E10.15	*snr-6*	83%	1%	3%	11%	2%	100
Y57A10A.19	*rsr-2*	92%	1%	2%	4%	1%	104
Y66D12A.8		91%	1%	3%	0%	5%	102
K06A5.7	*cdc-25.1*	91%	2%	2%	1%	5%	108

Gonad arms were scored in young adults using DIC microscopy.

a Gonad arms developed normally and migrated along the dorsal basement membrane and ceased opposite the vulva.

b Gonad arms failed to turn back to the midpoint of the animal, or took an extra turn during development.

c Gonad arms did not travel along the dorsal basement membrane or they displayed a “nose-dive” phenotype towards the vulva.

d Gonad arms continued to migrate along the dorsal basement membrane past the midpoint.

e Gonad arms stopped migrating prior to the vulva, gonad arms did not possess an intact structure or failed to develop.

## Discussion

In this study we isolate and characterize proteins associated *in vivo* with the *C. elegans* protein CACN-1. *cacn-1* is a highly conserved gene that is required for normal DTC migration and gonad morphogenesis (Tannoury *et al.* 2010). Consistent with previous results (Jurica *et al.* 2002; Ilagan *et al.* 2009; Bessonov *et al.* 2008, 2010; Baldwin *et al.* 2013; Ashton-Beaucage *et al.* 2014) this study provides evidence that CACN-1 interacts with spliceosomal proteins in *C. elegans*. The majority of proteins that copurified with CACN-1 are either known to regulate splicing in *C. elegans*, or have homologs associated with the human spliceosome. Because CACN-1 does not possess any conserved catalytic or RNA binding domains, we hypothesize that CACN-1 is acting as a scaffold for these spliceosomal components. Results from RNAi depletion experiments identify eight new regulators of DTC migration and suggest that these proteins may functionally interact with CACN-1 to regulate cell migration during *C. elegans* development.

In addition to a general requirement for the spliceosome in pre-mRNA processing, many accessory subunits are needed for specific alternative splicing events. Approximately 25% of all *C. elegans* transcripts are regulated by alternative splicing (Ramani *et al.* 2011), increasing the repertoire of proteins encoded by the genome and providing an additional mechanism for regulation of gene expression. For example, intron inclusion and subsequent nonsense-mediated decay is an important post-transcriptional mechanism for silencing gene expression (Lewis *et al.* 2003). Alternative splicing also enables expression of developmental stage-specific isoforms. For example, *let-2*, a collagen and component of the basement membrane found between the muscle and hypodermis, undergoes a dramatic transition of isoform expression between larval stages and adulthood (Sibley *et al.* 1993). Similarly, expression of specific protein isoforms may be required for each phase of DTC migration. The major defect we observed, failure to stop migrating at the end of L4, is consistent with failure of the DTC to convert from a larval (migratory) to an adult (stationary) phenotype. Isoforms specific to each phase of DTC migration have not been identified, but represent an important area for future research.

In mammalian systems, alternative splicing is an important regulator of cell migration, including regulation of the epithelial-mesenchymal transition (EMT) (Aparicio *et al.* 2013; De Craene and Berx 2013). EMT is the process in which an epithelial cell with apico-basolateral polarity is transformed to a cell that displays an invasive, migratory phenotype. Although this transition is a normal process in organismal development, it is also a hallmark of metastatic cancers (Han *et al.* 2013). EMT-associated splicing events are regulated by a variety of classes of splicing factors, and deregulation of these factors may lead to EMT transition and tumor metastasis (Shapiro *et al.* 2011). A broad range of gene classes relevant to EMT are affected by alternative splicing, (Shapiro *et al.* 2011) including genes involved in cytoskeletal regulation, cell motility, transforming growth factor-β signaling and the Wnt signaling network (Blencowe 2006; Sabbah *et al.* 2008; Wang *et al.* 2008; Polyak and Weinberg 2009).

In *C. elegans*, proteins with homology to splicing factors commonly also function as RNA binding proteins to regulate gene expression posttranscriptionally (Tamburino *et al.* 2013). Binding of mRNA, particularly at the 3′-untranslated region, prevents translation and stores the transcript until the protein is needed (Wilkie *et al.* 2003). For example, the splicing factor PRP-17 is also a posttranscriptional regulator of key germ line developmental genes (Kerins *et al.* 2010). In addition, splicing factor homologs have also been shown to play important roles in transcription, including direct interactions with RNA polymerase II (Chanarat *et al.* 2011; Fontrodona *et al.* 2013). Separate from the DTC migration phenotype, loss of CACN-1 function also causes defects in germline development (Kerins *et al.* 2010). Therefore, some of the components of the CACN-1 network may be working as both spliceosomal components and separately, as mRNA regulatory proteins and/or transcriptional regulators. This may represent a second important mechanism by which CACN-1 coordinates post-transcriptional regulation of gene expression.

Cactin homologs are well-conserved in all metazoans, and evidence from our work and others (Jurica *et al.* 2002; Bessonov *et al.* 2008, 2010; Ilagan *et al.* 2009) suggests that CACN-1 is a component of the spliceosome. Because of the universal importance and strong conservation of the spliceosome among metazoans, further elucidation of mRNA splicing in the worm should provide valuable insight into post-transcriptional gene regulation in humans and other animals and allow a more complete understanding of the mechanisms underlying cell migration.

## Supplementary Material

Supporting Information
