# Supplementary material for: Proteomic Analysis Reveals CACN-1 Is a Component of the Spliceosome in Caenorhabditis elegans
Source: G3 (Bethesda). 2014 Jun 19;4(8):1555–64. doi: 10.1534/g3.114.012013 (PMC4132184; doi:10.1534/g3.114.012013)
Supplement: Supporting Information [file supp_4_8_1555__index.html]

Proteomic Analysis Reveals CACN-1 Is a Component of the Spliceosome in Caenorhabditis elegans — Supporting Information 

# Proteomic Analysis Reveals CACN-1 Is a Component of the Spliceosome in *Caenorhabditis elegans*

## Supporting Information for Doherty *et al.*, 2014

**Files in this Data Supplement:**

- Table S1 - Proteins corresponding to peptides co-purified with HA::FLAG::CACN-1 data derived from Wormbase WS241. (PDF, 177 KB)
