# Supplementary material for: Proteomic Analysis Reveals CACN-1 Is a Component of the Spliceosome in Caenorhabditis elegans
Source: G3 (Bethesda). 2014 Jun 19;4(8):1555–64. doi: 10.1534/g3.114.012013 (PMC4132184; doi:10.1534/g3.114.012013)
Supplement: Supporting Information [file supp_g3.114.012013_012013SI.pdf]

G3 · Genes | Genomes | Genetics

Proteomic analysis reveals CACN-1 is a component of the spliceosome in *C. elegans*

Michael F. Doherty[*], Guillaume Adelmant[§,**] , Alyssa D. Cecchetelli[*], Jarrod A. Marto[§,**] Erin J. Cram[*,1]

[*] Biology Department, Northeastern University, Boston, Massachusetts, 02115
[§] Department of Cancer Biology and Blais Proteomics Center, Dana-Farber Cancer Institute, Boston, Massachusetts, 02215
[**]Department of Biological Chemistry and Molecular Pharmacology, Harvard Medical School, Boston, Massachusetts, 02115

Erin J. Cram[1]: Biology Department, Northeastern University, 360 Huntington Avenue, Boston, Massachusetts, 02115
Email: e.cram@neu.edu
Phone: 617-373-7753

DOI: 10.1534/g3.114.012013

Table S1 Proteins corresponding to peptides co-purified with HA::FLAG::CACN-1 data derived from Wormbase WS241

Locus	Gene ID	Gene Symbol
CE20137 WBGene00012230 locus:cacn-1 status:Partially_confirmed UniProt:Q9N6M0 protein_id:CAB76745.1	175066	cacn-1
CE00122 WBGene00004187 locus:prp-8 status:Partially_confirmed UniProt:P34369 protein_id:AAA27977.1	176153	prp-8
CE40670 WBGene00001258 locus:emb-4 status:Partially_confirmed UniProt:Q9U1Q7 protein_id:CAB60444.4	180232	emb-4
CE09682 WBGene00002005 locus:hsp-1 HSP-1 heat shock 70kd protein A status:Confirmed UniProt:P09446 protein_id:CAB02319.1	178507	hsp-1
CE28622 WBGene00019762 status:Partially_confirmed UniProt:O16376 protein_id:AAB65909.2	178979	M03F8.3
CE05066 WBGene00001166 locus:eftu-2 elongation factor 2 status:Confirmed UniProt:Q23463 protein_id:AAA91248.1	175851	eftu-2
CE06519 WBGene00004806 locus:skp-1 Drosophila puff specific protein BX42 like status:Confirmed UniProt:Q22836 protein_id:CAA98552.1	179598	skp-1
CE28405 WBGene00018625 locus:prp-17 status:Confirmed UniProt:O44729 protein_id:AAB96707.2	172999	prp-17
CE25503 WBGene00013260 locus:rsr-2 status:Partially_confirmed UniProt:Q9U213 protein_id:CAB60765.1	174862	rsr-2
CE05540 WBGene00008386 MYB transforming protein status:Confirmed UniProt:Q21119 protein_id:CAB00038.1	172639	D1081.8
CE08909 WBGene00016837 status:Partially_confirmed UniProt:P91175 protein_id:AAB37794.1	171970	C50F2.3
CE01889 WBGene00003393 locus:mog-5 RNA helicase status:Confirmed UniProt:Q09530 protein_id:AAC46765.1	173920	mog-5
CE24152 WBGene00000889 locus:cyn-13 status:Confirmed UniProt:Q9U2S6 protein_id:CAB55151.1	178487	cyn-13
CE38104 WBGene00020423 status:Confirmed UniProt:Q10051 protein_id:AAK21467.2	175705	T10F2.4
CE28806 WBGene00013434 status:Confirmed UniProt:Q95Q06 protein_id:CAC70131.1	176589	Y66D12A.8
CE07669 WBGene00004415 locus:rpl-4 ribosomal protein L1 status:Confirmed UniProt:O02056 protein_id:AAC24253.1	172074	rpl-4
CE18578 WBGene00008586 WD domain, G-beta repeat (6 domains) status:Confirmed UniProt:Q19211 protein_id:CAA91460.1	181319	F08G12.2

CE03921 WBGene00006929 locus:vit-5 status:Confirmed UniProt:P06125 protein_id:AAA83587.1	180630	vit-5
CE22002 WBGene00002016 locus:hsp-16.2 heat shock protein status:Confirmed UniProt:P06582 protein_id:AAF60615.1	178659	hsp-16.2
CE30781 WBGene00004450 locus:rpl-36 Ribosomal protein YL39 status:Confirmed UniProt:P49181 protein_id:AAC48295.2	176007	rpl-36
CE00664 WBGene00004470 locus:rps-1 Ribosomal protein S3a (human) homolog. status:Confirmed UniProt:P48154 protein_id:CAA83605.1	175584	rps-1
CE04561 WBGene00004477 locus:rps-8 40S ribosomal protein S8 status:Confirmed UniProt:P48156 protein_id:AAA81485.1	177503	rps-8
CE00854 WBGene00004469 locus:rps-0 40S ribosomal protein status:Confirmed UniProt:P46769 protein_id:CAA86061.1	175628	rps-0
CE20974 WBGene00044076 locus:sap-1 status:Confirmed UniProt:Q9BLB6 protein_id:AAK31542.1	173767	sap-1
CE14251 WBGene00002019 locus:hsp-16.48 heat shock protein HSP16-48 status:Predicted UniProt:P02513 protein_id:AAB04841.1	179288	hsp-16.48
CE22230 WBGene00004919 locus:snr-6 status:Confirmed UniProt:Q9XTU6 protein_id:CAB11551.1	176668	snr-6
CE20938 WBGene00006725 locus:ubl-1 ubiquitin status:Confirmed UniProt:P37165 protein_id:AAF39865.1	175413	ubl-1
CE08110 WBGene00002026 locus:hsp-70 heat shock protein 70 status:Partially_confirmed UniProt:O45246 protein_id:CAB03871.1	172757	hsp-70
CE13265 WBGene00004488 locus:rps-19 Ribosomal protein S19e status:Confirmed UniProt:O18650 protein_id:CAB04689.1	172805	rps-19
CE04746 WBGene00006925 locus:vit-1 vit-1 status:Confirmed UniProt:P55155 protein_id:AAB52675.1	181034	vit-1
CE05922 WBGene00009966 status:Confirmed UniProt:Q20716 protein_id:CAA96652.1	179524	F53B7.3
CE26774 WBGene00021350 status:Confirmed UniProt:Q9BKU5 protein_id:AAK27870.1	171767	Y37E3.8
CE09655 WBGene00004408 locus:rla-0 deoxyribonuclease status:Confirmed UniProt:Q93572 protein_id:CAB02098.1	172943	rla-0
CE05721 WBGene00004439 locus:rpl-25.2 60S ribosomal protein status:Confirmed UniProt:Q20647 protein_id:CAA99858.1	172617	rpl-25.2
CE33830 WBGene00011722 RNA recognition motif. (aka RRM, RBD, or RNP domain) status:Confirmed UniProt:Q22412 protein_id:CAA93419.2	177979	T11G6.8
CE17321 WBGene00002191 locus:kin-3 casein kinase status:Confirmed UniProt:P18334 protein_id:AAC16993.1	172978	kin-3

CE04713 WBGene00006351 locus:sur-5 acetyl-coenzyme A synthetase status:Confirmed UniProt:Q21166 protein_id:AAA83327.1	180992	sur-5
CE26217 WBGene00013344 locus:ebp-1 status:Partially_confirmed UniProt:Q9GRZ1 protein_id:CAC14408.1	3565059	ebp-1
CE01697 WBGene00014250 YJU2 protein status:Confirmed UniProt:Q09651 protein_id:CAA87435.1	174518	ZK1307.9
CE08034 WBGene00004431 locus:rpl-19 60S ribosomal protein L19 status:Confirmed UniProt:O02639 protein_id:AAB53979.1	172201	rpl-19
CE14030 WBGene00020718 ribosomal protein status:Confirmed UniProt:O17004 protein_id:AAB69935.1	179229	T23B12.3
CE05598 WBGene00004414 locus:rpl-3 60S ribosomal protein L3 status:Confirmed UniProt:P50880 protein_id:CAA91277.1	175501	rpl-3
CE06580 WBGene00001337 locus:ears-1 glutamyl-trna synthetase status:Confirmed UniProt:Q23315 protein_id:CAB00060.1	172904	ears-1
CE09945 WBGene00004484 locus:rps-15 40S ribosomal protein S15 status:Confirmed UniProt:Q9XVP0 protein_id:CAB03065.1	172693	rps-15
CE00778 WBGene00004435 locus:rpl-23 status:Confirmed UniProt:P48158 protein_id:AAK18857.1	175796	rpl-23
CE29835 WBGene00004489 locus:rps-20 status:Confirmed UniProt:Q8WQA8 protein_id:CAD21665.1	173309	rps-20
CE25552 WBGene00004412 locus:rpl-1 status:Confirmed UniProt:Q9N4I4 protein_id:AAF36008.1	171853	rpl-1
CE00499 WBGene00007400 G10 protein status:Confirmed UniProt:P34313 protein_id:CAA82338.1	176368	C07A9.2
CE22003 WBGene00002018 locus:hsp-16.41 heat shock protein status:Confirmed UniProt:P06581 protein_id:AAF60616.1	178660	hsp-16.41
CE31498 WBGene00009439 locus:mlcd-1 malonyl-COA decarboxylase status:Confirmed UniProt:Q20048 protein_id:CAA86324.2	175597	mlcd-1
CE14734 WBGene00004076 locus:pod-2 acetyl-CoA carboxylase status:Partially_confirmed UniProt:Q9GZI3 protein_id:AAG00029.1	173500	pod-2
CE09162 WBGene00008505 phosphate carrier protein precursor status:Confirmed UniProt:P40614 protein_id:CAA97430.1	178020	F01G4.6
CE04286 WBGene00000884 locus:cyn-8 status:Confirmed UniProt:P52016 protein_id:AAA81696.1	181136	cyn-8
CE30433 WBGene00016493 ribosomal protein status:Confirmed UniProt:O01504 protein_id:AAB52450.2	172401	C37A2.7
CE04691 WBGene00004494 locus:rps-25 ribosomal protein status:Confirmed UniProt:P52821 protein_id:AAK39246.1	177365	rps-25

Data derived by Wormbase WS241
